# The role of circRNAs in cancers

**DOI:** 10.1042/BSR20170750

**Published:** 2017-10-24

**Authors:** Ling-Ping Zhu, Yun-Jie He, Jun-Chen Hou, Xiu Chen, Si-Ying Zhou, Su-Jin Yang, Jian Li, He-Da Zhang, Jia-Hua Hu, Shan-Liang Zhong, Jian-Hua Zhao, Jin-Hai Tang

**Affiliations:** 1 Department of General Surgery, The First Clinical School of Nanjing Medical University, Nanjing 210029, P.R. China; 2 Department of General Surgery, The First Affiliated Hospital with Nanjing Medical University, Nanjing 210029, P.R. China; 3 Department of General Surgery, The First Clinical School of Nanjing University of Chinese Medicine, Nanjing, China; 4 Department of General Surgery, Nanjing Medical University Affiliated Cancer Hospital Cancer Institute of Jiangsu Province, Baiziting 42, Nanjing 210009, China; 5 Department of General Surgery, Southeast University Medical School, Nanjing, Jiangsu, China; 6 Center of Clinical Laboratory, The Fourth Clinical School of Nanjing Medical University, Baiziting 42, Nanjing 210009, China; 7 Center of Clinical Laboratory, Nanjing Medical University Affiliated Cancer Hospital Cancer Institute of Jiangsu Province, Baiziting 42, Nanjing 210009, China

**Keywords:** Biomarker, Circular RNAs, Cancer, Function

## Abstract

Circular RNAs (circRNAs) are recently regarded as a naturally forming family of widespread and diverse endogenous noncoding RNAs (ncRNAs) that may regulate gene expression in mammals. At present, above 30000 circRNAs have already been found, with their unique structures to maintain stability more easily than linear RNAs. Several previous literatures stressed on the important role of circRNAs, whose expression was relatively correlated with patients’ clinical characteristics and grade, in the carcinogenesis of cancer. CircRNAs are involved in many regulatory bioprocesses of malignance, including cell cycle, tumorigenesis, invasion, metastasis, apoptosis, vascularization, through adsorbing RNA as a sponge, binding to RNA-binding protein (RBP), modulating transcription, or influencing translation. Therefore, it is inevitable to further study the interactions between circRNAs and tumors and to develop novel circRNAs as molecular markers or potential targets, which will provide promising applications in early diagnosis, therapeutic evaluation, prognosis prediction of tumors and even gene therapy for tumors.

## Introduction

Besides the conventional coding mRNA, tRNA, and rRNAs, there is a tremendous diversity of noncoding attached RNA genres which exist in the cells, such as miRNAs, lncRNAs, piRNAs, siRNAs, tmRNAs, sRNAs, tiRNAs, eRNAs, snoRNAs, snRNAs, and other noncoding RNAs (ncRNAs). While an indispensable compose of this diverse RNA molecules family is the circular RNAs (circRNAs) whose detected amounts and types is increasing at an accrescent rate.

CircRNAs are a wide category of ncRNAs that are verified to participate in regulating transcriptional and post-transcriptional gene expression [1]. In 1976, Sanger et al. [2] who first presented the concept of ‘circular RNA’ found that viroids are single-stranded covalently closed circRNA molecules pathogenic to certain higher plants. Subsequently, circRNAs were clearly observed in the eukaryotes by electron microscopy in 1979 [3]. Even so, circRNAs were regarded as a by-product of errant splicing due to their low abundance and lack of known functions [4–6]. With the development of high-throughput RNA sequencing and bioinformatics analysis, more and more circRNAs have been discovered and identified [7–9]. Beyond gene regulation, circRNAs can represent as potential biomarkers which may play new roles in cancer diagnosis and targetted therapy.

## Characteristics of circRNAs

**Figure 1:**
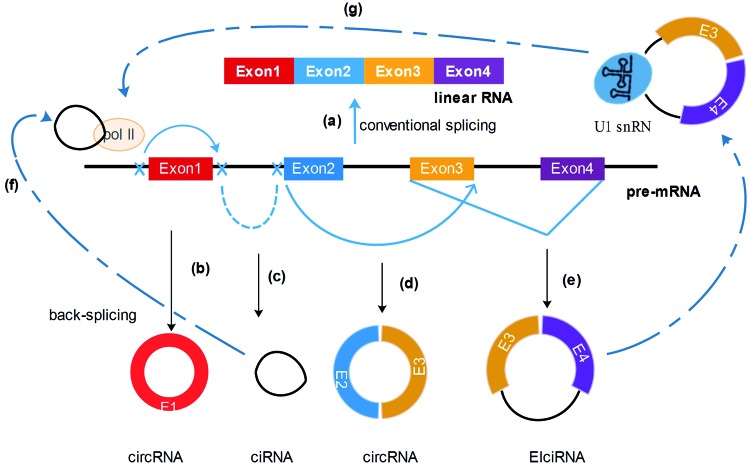
Schematic representation of splicing events and biological functions of circRNAs (**a**) Linear mRNA is generated conventionally through canonical splicing machinery. (**b**) Exonic circRNA is formed through backsplicing of the 5′ splice site (donor site) to a 3′ splice site (acceptor site) which is called head-to-tail joining. (**c**) Reverse complementary sequences of lariat intron excised from pre-mRNA can pair to produce close loop structure named as ciRNA. (**d**) The intron2 is then removed and brings the 5′ splice site of Exon3 close to 3′ splice site of Exon2, to form a circRNA, which contains multiple exons. (**e**) Also, intron3 will be retained, with Exon3 and Exon4, forming an EIciRNA. (**f**) The stable ciRNA binds to elongating RNA Pol II and promotes transcription. (**g**) EIciRNAs can enhance gene transcription via interacting with U1 snRNP and RNA polymerase II in the promoter region of the host gene.

**Figure 2:**
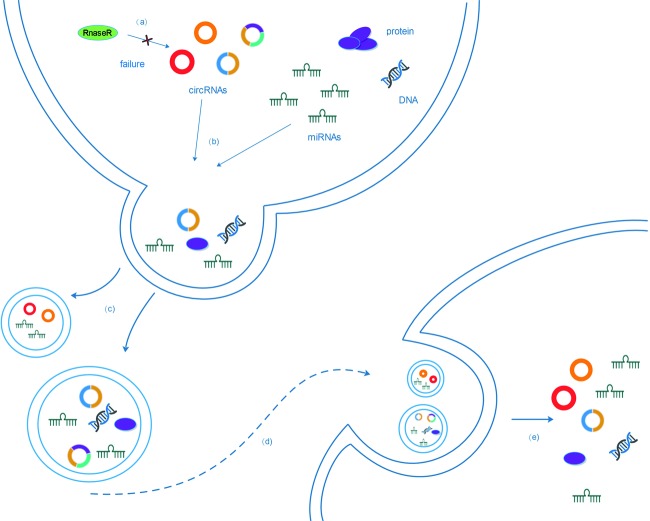
The expulsion and transport of circRNAs (**a**) Without 5′ caps and 3′ tails, circRNAs which can resist degradat by RNase R, are highly stable. (**b**,**c**) Extracellular vesicles can carry a myriad of cellular components, including proteins, lipids, and RNA, despite their small size. Also, extracellular vesicle release may be one of the ways for cells to eliminate circRNAs. (**d**,**e**) Exosomes release the endocytosed contents to the external environment or distant target cells, to transfer biological signals through one cell to another.

Many researches indicated that circRNAs were of great diversities for its generation originated from any region of the genome subsequence [9–14]. Amongst them, a large proportion of circRNAs arise from only one exon or multiple amounts of exons [15–18] (Figure 1). The exonic circRNAs predominantly remain in the cytoplasm [19,20]. Another subset of circRNAs known as EIciRNAs (exon–intron circRNAs) exists, which contains both exons and introns [13,21]. Moreover, lariat intron’s failure to debranch at the branch point site and the trimming of the lariat tail lead to the formation of circular intronic RNAs (ciRNAs), mainly found in the nucleus [13,22] (Figure 1). Unlike linear RNAs, which are directly terminated with 5′ caps and 3′ tails, circRNAs are formed as a covalently closed continuous loop by 5′ splice site (the splice donor) joined to a 3′ splice site (the splice acceptor) which is called backsplicing [23,18,24,14,5] (Figure 1). Hence, without 5′–3′ polarity and polyadenylated tail, circRNAs are more stable than liner RNA and can resist degradation by RNA exonuclease or RNase R [7,25] (Figure 2). In recent decades, many studies revealed that circRNAs were abundant in mammals with accumulation in a tissue-specific manner, especially enriched in synaptoneurosomes [7,19,8,26,27]. Furthermore, a research showed that circRNAs were enriched in exosomes compared with the producer cells [28]. Extracellular vesicles can carry plenty of cellular components, including RNA, DNA, proteins, and lipids, then release the endocytosed contents to the external environment or distant target cells [29–31] (Figure 2). In another words, due to the vesicle’s insolated role from outer conditions in compliance with its resistance to major mRNA degrading enzymes, the release of extracellular vesicles may be one of the ways for cells to eliminate circRNAs or to transfer biological signals through one cell to another [32] (Figure 2).

So, there are approximately three types of circRNAs, and they are more stable than linear RNAs. Their capacity in tumor cells could be modulated by exosome transportations or some kinds of other ways.

## The function of circRNAs

**Figure 3:**
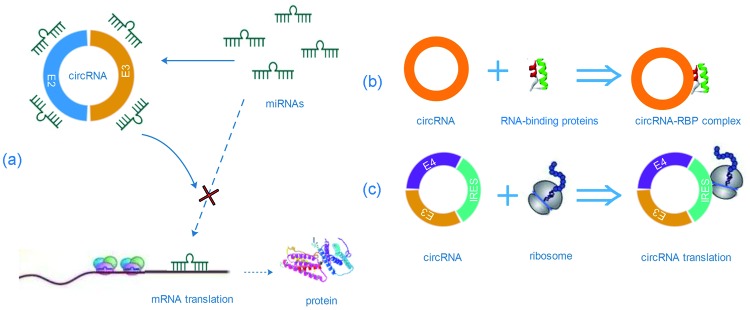
The biological functions of circRNA (**a**) CircRNAs act as miRNA sponge to compete endogenous RNA and sequester miRNAs from binding mRNA targets to influence the protein translation. (**b**) CircRNAs can also work as RBP sponge to interact with RBPs, forming RNA–protein complex (RPC). (**c**) The synthetic circRNA which contains an internal ribosome entry site (IRES) can be translated to produce proteins *in vitro*.

Currently, the circRNA has become a hotspot in the field of biology on account of its huge capabilities. Studies have found that circRNAs can bind to miRNA as RNA sponge and increase downstream gene expression by regulating miRNA activities, thus contributing to tumor progression [33,34,1,21] (Figure 3). For example, the earliest circRNA CiRS-7 contains more than 70 *miR-7* binding sites and acts as an *miR-7* sponge, reducing the impact of *miR-7* on target mRNAs in turn [35,36,1]. Lately, a report demonstrated that cir-ZNF609 might act as a sponge for *miR-150-5p* to modulate the expression of AKT3 [37]. In addition, circRNAs are also involved in transcription, translation, splicing, and RNA-binding proteins (RBPs) [17,38,39,12] (Figures 1 and 3). Interestingly, circRNAs may interact with other RNA molecules such as mRNAs and lncRNAs, or even with DNA directly [40,41]. In addition, ciRNA binds to elongating RNA Pol II and promotes transcription in turn. EIciRNAs can interact with U1 snRNP and RNA Polymerase II in the promoter region of the host gene to enhance gene transcription as well [12] (Figure 1).

Moreover, some studies have reported that circRNAs were concerned with many kinds of human diseases, and were widely involved in numerous physiological and pathological processes, for instance promoting insulin biosynthesis and secretion through the CDR1as/*miR-7* pathway, and dysregulation in Alzheimer’s disease (AD), Parkinson’s disease, and cardiovascular diseases [36,42–44]. Importantly, some findings indicated that dysregulated circRNAs had been verified to be associated with the development of many cancers [45,46] (Table 1).

**Table 1:** The connection between circRNAs and human cancers

Type of cancer	circRNA	Bind target	Function(s)	References
**Gastric cancer**	Hsa_circ_002059			[47]
	hsa_circ_0000096	Regulating cyclin D1, CDK6, MMP-2, and MMP-9	Cell growth and migration	[48]
	circPVT1	*miR-125*	Promotes cell proliferation	[49]
	hsa_circ_0001649			[50]
	hsa_circ_0000069			[51]
**Colorectal cancer**	hsa_circ_001569	*miR-145*	Cell proliferation and invasion, differentiation and invasion facilitates proliferation	[52]
	hsa_circ_001988			[53]
	circ-BANP			[54]
	CiRS-7	*miR-7*		[55]
**Hepatocellular carcinoma**	hsa_circ_0005075	*miR-23b-5p, miR-93-3p, miR-581, miR-23a-5p*	Cell adhesion	[57]
	CircZKSCAN1	*ZKSCAN1* mRNA	Inhibits growth, migration, invasion	[58]
**Bladder carcinoma**	circTCF25	*miR-103a-3p/miR-107*	Increases CDK6 expression	[74]
**Esophageal cancer**	hsa_circ_0067934		Promotes the proliferation	[60]
	cir-ITCH	*miR-7, miR-17, miR-214*	Stimulates ITCH and suppresses	[61]
			Wnt/β-catenin pathway	
**Cervical carcinoma**	hsa_circ_0031288	HuR	sequester HuR from PABPN1 mRNA and reduce translation	[62]
**Breast cancer**	circ-Foxo3	p21 and CDK2	Inhibits cell proliferation and cell cycle progression	[38]
**Laryngeal cancer**	hsa_circRNA_104912		Participates in the tumorigenesis	[66]

Abbreviations: CDK2, cyclin-dependent kinases2; CDK6, cyclin-dependent kinases2; circ-BANP, circular BANP; Foxo3, forkhead box O3; HuR, ELAV-like RBP 1; ITCH, itchy E3 ubiquitin protein ligase; MMP-2, matrix metalloprotein-2; MMP-9, matrix metalloprotein-9; PABPN1, poly(A) binding protein nuclear 1; TCF25, transcription factor 25; ZKSCAN1, a zinc finger family gene.

In conclusion, circRNAs take part in the biological functions of tumors mainly by kinds of approaches to react with other RNA genres or genes.

### Gastric cancer

Hsa_circ_002059, a typical circRNA, was first found to be significantly down-regulated in gastric cancer (GC) tissues compared with paired adjacent nontumorous tissues [47]. Lately, hsa_circ_0000096 was also found to be markedly down-regulated in both GC tissues and GC cell lines relative to the control paired adjacent nontumorous tissues and normal gastric epithelial cells, which may attenuate GC cell growth and migration by regulating cyclin D1, cyclin-dependent kinases6 (CDK6), matrix metalloprotein-2 (MMP-2), and matrix metalloprotein-9 (MMP-9) [48]. The expression of circPVT1 in GC tissues was often up-regulated, and functional assays revealed that circPVT1 could promote cell proliferation by sponging members of *miR-125* family. It can be also an independent prognostic marker for survival in patients with Gc [49]. Another circRNA called hsa_circ_0001649 presented a lower expression in GC tissue than the control normal tissues. Besides, the expression level of hsa_circ_0001649 in patients’ serum samples would be meaningfully increased after the tumor resected surgery [50]. It suggested that many circRNAs possessed significantly differential expressions between GC tissues and normal tissues, and the circRNA may become a novel potential and stable biomarker in the diagnosis of gastric carcinoma.

### Colorectal cancer

He et al. found that hsa_circ_0000069 had a significantly high expression in colorectal cancer (CRC) tissues and that it correlated positively with the degree of clinical features, such as the TNM stage [51]. A high expression of hsa_circ_001569 was also observed in CRC tissues, which could act as a sponge to bind to *miR-145*, and resulted in the up-regulation of target genes of *miR-145*, including E2F transcription factor 5 (E2F5), BCL2-associated athanogene 4 (BAG4), and formin like 2 (FMNL2), thus playing as a positive regulator in cell proliferation and invasion of the CRC [52]. Even more, it has also been suggested that the expression of hsa_circ_001988 was meaningfully decreased in tumor tissues analyzed from 31 matched CRC tissue and normal colon mucosa by quantitative real-time PCR (qRT-PCR). Moreover, it significantly connected with differentiation and perineural invasion [53]. Also, evidence detected that circular BANP (circ-BANP) differential expressions by analyzing circRNA array of both CRC cancerous tissues and adjacent normal tissues, which confirmed the facilitation of the proliferation in CRC cells. Meanwhile, the growth of cancer cells will be significantly deadened after knockdown of circ-BANP with siRNA [54]. Thus, it can be seen that the dysregulation of circRNAs may participate in cell proliferation, differentiation, and invasion in CRC.

### Hepatocellular carcinoma

As we all know, *miR-7* is able to inhibit tumor growth and metastasis by targetting the phosphoinositide 3-kinase/Akt (PI3k/AKT) pathway in hepatocellular carcinoma (HCC) [55]. While CiRS-7 may play a significant role in the development of HCC by acting as a sponge to bind to *miR-7* [56]. Furthermore, Shang et al. [57] discovered that hsa_circ_0005075 exhibited a significant difference in expressions between HCC and normal tissues through using real-time qRT-PCR. Then, they structured a network of hsa_circ_0005075-targetted miRNA–gene interactions, including *miR-23b-5p, miR-93-3p, miR-581, miR-23a-5p*, and their corresponding targetted mRNAs. Gene oncology analysis revealed that hsa_circ_0005075 could participate in cell adhesion during HCC development [57]. CircZKSCAN1, originated from a zinc finger family gene (ZKSCAN1), was discovered with significantly lower expression in 102 HCC patients by RT-PCR, and the levels influenced in patients by different tumor numbers, cirrhosis, vascular invasion, or microscopic vascular invasion (MVI), as well as the tumor grade, which could co-operate closely with *ZKSCAN1* mRNA to inhibit growth, migration, and invasion of HCC [58]. In conclusion, circRNAs could regulate directed mRNAs via adsorbing correlative miRNAs or both work in co-ordination to control the growth, migration, and invasion of HCC.

### Bladder carcinoma

Zhong et al. detected circRNAs with prominent differential expression using microarray analysis in samples of bladder cancer. Compared with the normal tissues, 469 dysregulated circular transcripts are found in total. Six circRNAs, including circTCF25 (transcription factor 25) (hsa_circ_0041103), were confirmed to have signifcant differences by qRT-PCR. Furthermore, it was verified that overexpression of circTCF25 could sequester *miR-103a-3p/miR-107* to suppress miRNA biological activity, consequently increase CDK6 expression, and promote proliferation and migration in bladder cancer [59]. As we have noticed, circRNAs are possibly involved in the pathogenesis and development of bladder cancer.

### Esophageal cancer

In 51 pairs of cancerous tissues and adjacent noncancerous tissues derived from esophageal cancer patients, hsa_circ_0067934 was detected to be conspicuously overexpressed in esophageal squamous cell carcinoma (ESCC) tissues and was associated with poor differentiation, T stage and TNM stage through qRT-PCR. Afterward, the siRNA designed to inhibit hsa_circ_0067934 was treated with ESCC cells, resulting in restraining the proliferation and migration of ESCC cells. Therefore, we hypothesized that hsa_circ_0067934 could promote the proliferation of ECSS cells by regulating the cell cycle [60]. Another circRNA named cir-ITCH (itchy E3 ubiquitin protein ligase), acquired from a total of 684 ESCC and paired adjacent nontumor tissue samples via RT-PCR, which could act as a sponge for *miR-7, miR-17*, and *miR-214*, had an inhibitory effect on ESCC by stimulating ITCH levels to promote an ubiquitin-mediated dishevelled segment polarity protein 2 (Dvl2) degradation, suppressing the canonical Wnt/β-catenin [61]. These results indicate that hsa_circ_0067934 may work as an emerging potential tumor marker and therapeutic target of ESCC and cir-ITCH can inhibit the evolution of ESCC by regulating the Wnt pathway.

### Cervical carcinoma

It was demonstrated that hsa_circ_0031288, renamed circPABPN1 (poly(A) binding protein nuclear 1) as it rose from the PABPN1 pre-mRNA, could bond with ELAV-like RBP 1 (HuR) to prevent HuR binding to *PABPN1* mRNA and reduce PABPN1 translation in human cervical carcinoma HeLa cells. While HuR influences gene expression programs and hence cellular phenotypes by binding to hundreds of coding and noncoding linear RNAs [62]. In other words, circPABPN1 could affect translation through combining with RBPs to sequester it from mRNA in HeLa cells.

### Breast cancer

Remarkably, a study has manifested that the overexpression of circ-Foxo3 (forkhead box O3) in MDA-MB-231 cells could restrain tumor growth *in vivo* as well as cancer cell proliferation and survival *in vitro* [63]. Further studies showed that circ-Foxo3 inhibited cell proliferation and repressed cell cycle progression by binding to p21 and cyclin-dependent kinases2 (CDK2), forming a ternary circ–Foxo3–p21–CDK2 complex [38]. Recently, a new data analysis of 885 breast cancer samples provided by The Cancer Genome Atlas (TCGA) revealed that the number of circRNAs had a relation to breast cancer subtypes, which in estrogen receptor positive (ER+) subtype of normal adjacent tissues was higher than tumor tissues using CircRNA-Seq, and was connected with gene proliferation markers [64]. Although the interaction mechanism between circRNAs and breast cancer awaits to be further explored, circRNAs certainly implicate in cell cycle progression in breast cancer.

### Epithelial ovarian carcinoma

In order to inquire into the effect of circRNAs in ovarian cancer, the paired-end RNA sequencing of nine ovarian cancer samples derived from three patients at primary ovarian tumor and its matched peritoneum and lymph node metastases, were implemented by Ahmed et al. [65], showing that a noteworthy greater number of circRNAs were differentially expressed between tumor sites than mRNAs. These candidate circRNAs are concentrated for multiple miRNA matching points and are possibly able to compete with endogenous RNA activity. Moreover, it was a robust expression pattern of circRNAs across patients and tumor stages, in contrast with a highly heterogeneous linear transcriptome in ovarian cancer [65]. The conformance of circRNA expression may afford fresh candidates for cancer treatment and prognosis.

### Laryngeal cancer

Xuan et al. [66] discovered that 302 circRNAs were signifcantly up-regulated and 396 circRNAs were down-regulated in four paired laryngeal squamous cell carcinoma (LSCC) tissues and adjacent nontumor tissues using microarray analysis. Results were further proved by qRT-PCR methods, revealing that hsa_circRNA_100855 was the most up-regulated circRNA and hsa_circRNA_104912 was the most down-regulated circRNA, and both were relevant to T3–4 stage, neck nodal metastasis, poor differentiation, or advanced clinical stage [66]. Overall, the data illustrate that circRNAs may relate to the tumorigenesis of LSCC and represent a new molecular marker for the diagnosis and progress of LSCC.

### CircRNAs in cancer-associated pathways

CircRNAs have attracted much attention in cancer research field. They are involved in the cellular development, proliferation, differentiation, and apoptosis. For example, circ-Foxo3 can bind to p21 and CDK2, and form a ternary circ–Foxo3–p21–CDK2 complex, inhibiting the function of CDK2 and arresting cell cycle progression [38,63]. While circHIPK3 (homeodomain interacting protein kinase 3) would influence the proliferation of tumor cells through genes, including protein phosphatase 1 regulatory subunit 13 like (iASPP) by working as the sponge of *miR-124* to inhibit *miR-124* activity [67,68]. Moreover, a study found that lncRpa and circRar1 could modulate *miR-671* for inducing the up-regulation of apoptosis-associated factors caspase8 and p38 at the mRNA and protein levels to promote neuronal apoptosis [69]. For example, the promyelocytic leukemia/ retinoic acid receptor α (PML/RARa) and lysine methyltransferase 2A (MLL) genes fused and then produced f-circM9 and f-circPR. While knockouts of f-circM9 and f-circPR led to the apoptosis of a large amount of tumor cells, thus the f-circM9 and f-circPR, to some extent, could abate cancer cell apoptosis [70]. For instance, cir-ITCH could act as the sponge of oncogenic *miR-7* and *miR-214* to enhance ITCH expression and thus suppress the activation of Wnt/β-catenin signaling [61]. Circular and linear expression exhibits an inverse trend for many cancer-related pathways and signaling pathways, like nuclear factor κB (NFκB), Janus kinase/signal transducer and activator of transcription (JAK/STAT), PI3k/AKT, and transforming growth factor β (TGF-β), which were typically activated for mRNA in metastases and down-regulated for circRNA [65]. Meanwhile, it was found that down-regulation of cZNF292 contributed to decreased transcription of E2F transcription factor 1 (E2F1), NF-κB, Sp1 transcription factor (Sp1), hypoxia inducible factor 1 (HIF-1), AP-1 transcription factor subunit (AP-1), signal transducer and activator of transcription 3 (STAT3), and signal transducer and activator of transcription 5 (STAT5), thus inhibiting the tube formation in tumor cells [71,72]. Thus, the dysregulation of circRNAs may be involved in tumorigenesis, progression, invasion, and metastasis in various cancers [73,58,74,52,51].

## CircRNAs may act as newly emerging biomarkers in cancer

As we all have noticed, with the application of RNA-seq and other detecting techniques, the expression difference of circRNAs are found to be widespread in many cancers, including esophageal cancer [60], GC [48], CRC [54], and HCC [55] etc. (Table 1). Besides, circRNAs are more steady, easier to extract, easily detected in clinical standard blood samples or body fluid, relative to linear RNAs and proteins [75–77]. CircRNAs originated from human cancer xenografts could release into the circulation and readily measured in the serum [28]. These features have a great clinical advantage for circRNAs to serve as novel biomarkers for diagnostic and therapeutic methods of cancers. Meanwhile, circRNAs play a potential critical role in contacting miRNA, mRNA, protein, and gene with each other in cancer cells [78,16,21]. Thus, the circRNA is an ideal molecular marker for cancer.

## Conclusion and perspective

Although the functions of most circRNAs are largely unclear, existing evidence has shown that circRNAs function in multiple biological processes, such as miRNA binding, RBPs binding, and regulation of transcription or splicing. Strikingly, the newest study also exhibited that circRNAs could be translated to produce proteins because it contains N6-methyladenosine (m6A) sites as internal ribosome entry site (IRES) (Figure 2), which were reduced by m6A demethylase FTO, α-ketoglutarate-dependent dioxygenase, promoted by adenosine methyltransferase (methyltransferase like 3/14 (METTL3/14)), and required eukaryotic translation initiation factor 4 γ 2 (eIF4G2) and m6A reader YTH N6-methyladenosine RBP 3 (YTHDF3) [79,80]. A general function of circRNA is suggested and has important implications in the translation landscape of human genome. For now, it appears that we are only beginning to catch a glimpse of the whole complexity of the regulatory mechanisms based on circRNAs. Thus, we must encourage research efforts to investigate the potential roles of circRNAs in translation that might contribute to the elucidation of gene function. Moreover, a great number of circRNAs were validated to be dysregulated in the CD28 (−) CD8 (+) T cells during ageing using a circRNA microarray approach. The pattern of a circRNA–miRNA–gene network was predicted, indicating that circRNA100783 might regulate phosphoprotein-related signal transduction in the context of CD28-dependent CD8(+) T-cell ageing [81]. Accordingly, circRNA also plays important roles in the regulation of immune system.

Additionally, more than 1000 circRNAs were identified in human serum exosomes. Surprisingly, it has been shown that circRNAs were concentrated in exosomes. Besides, the sorting of circRNAs to exosomes may be regulated by changes in associated miRNA levels in producer cells, and may transfer biological activities to recipient cells. Furthermore, circRNAs stored in exosomes still retained biological activities and could sequester miRNAs in receipt cells. It can be deduced that circRNAs contained in exosomes (exo-circRNAs) originated from human cancer xenografts enter the circulation and are easily measured in the serum, and also can be taken by target cells, leading to the change in tumor microenvironment and distant organ metastasis. In conclusion, it demonstrates that abundant circRNAs exist in the exosomes, representing a specific class of stable RNA species in exosomes, which can distinguish patients with cancer from the healthy and may act as a circulating biomarker for cancer diagnosis and prognosis. At present, recent researches have mainly focussed on the mechanisms of circRNA biogenesis and basic functions in primary tumor cells. However, a deeper mechanism in the exosomes and the developments of diverse diseases are not yet fully understood. Furthermore, it would be of great interest to understand the biological functions of exo-circRNAs and find other potential applications in future studies.

These findings greatly extended our knowledge of the abstruse complexity of gene regulation and have pushed circRNAs to the burning forefront of biological researches. Moreover, many databases, which will be more perfect, such as circBase, circNet, Circ2Traits, and CircInteractome, provided excellent platforms to further facilitate functional researches on circRNAs [82–84]. However, current studies on the relationship between circRNAs and disease physiology and pathology are limited. Taken together, although circRNAs are properly prevalent at present, the field still remains in its immature period. Further intensive studies are needed to explore the molecular and biological functions of circRNAs. CircRNAs, as the new star of ncRNA, would be widely involved in the regulation of physiological and pathophysiological processes, serving as stable clinical biomarkers of disease, and also provide new potential therapeutic targets. Exploration of their functions will bring brilliant prospects to mankind, further approaches to the essence of life.

## Informed consent

Informed consent was obtained from all the individual participants included in the study.

## Abbreviations

CDK2: cyclin-dependent kinases2
CDK6: cyclin-dependent kinases6
ciRNA: circular intronic RNA
circ-BANP: circular BANP
circRNA: circular RNA
CRC: colorectal cancer
EIciRNA: exon–intron circRNA
ESCC: esophageal squamous cell carcinoma
exo-circRNA: circRNAs contained in exosome
Foxo3: forkhead box O3
FTO: α-ketoglutarate-dependent dioxygenase
GC: gastric cancer
HCC: hepatocellular carcinoma
HuR: ELAV-like RNA-binding protein 1
ITCH: itchy E3 ubiquitin protein ligase
LSCC: laryngeal squamous cell carcinoma
MLL: lysine methyltransferase 2A
m6A: N6-methyladenosine
ncRNA: noncoding RNA
NFκB: nuclear factor κB
PABPN1: poly(A) binding protein nuclear 1
PI3k/AKT: phosphoinositide 3-kinase/Akt
qRT-PCR: quantitative real-time PCR
RBP: RNA-binding protein
TCF25: transcription factor 25
ZKSCAN1: a zinc finger family gene
